# Pharmacological Properties and Health Benefits of Eugenol: A Comprehensive Review

**DOI:** 10.1155/2021/2497354

**Published:** 2021-08-03

**Authors:** Muhammad Farrukh Nisar, Mahnoor Khadim, Muhammad Rafiq, Jinyin Chen, Yali Yang, Chunpeng Craig Wan

**Affiliations:** ^1^Jiangxi Key Laboratory for Postharvest Technology and Nondestructive Testing of Fruits & Vegetables, College of Agronomy, Jiangxi Agricultural University, Nanchang 330045, China; ^2^Department of Physiology and Biochemistry, Cholistan University of Veterinary and Animal Sciences (CUVAS), Bahawalpur 63100, Pakistan; ^3^College of Materials and Chemical Engineering, Pingxiang University, Pingxiang 330075, China; ^4^Department of Pathology, Affiliated Hospital of Yunnan University/Second People's Hospital of Yunnan Province, Kunming 650021, China

## Abstract

The biologically active phytochemicals are sourced from edible and medicinally important plants and are important molecules being used for the formulation of thousands of drugs. These phytochemicals have great benefits against many ailments particularly the inflammatory diseases or oxidative stress-mediated chronic diseases. Eugenol (EUG) is a versatile naturally occurring molecule as phenolic monoterpenoid and frequently found in essential oils in a wide range of plant species. EUG bears huge industrial applications particularly in pharmaceutics, dentistry, flavoring of foods, agriculture, and cosmeceutics. It is being focused recently due to its great potential in preventing several chronic conditions. The World Health Organization (WHO) has declared EUG as a nonmutant and generally recognized as safe (GRAS) molecule. The available literature about pharmacological activities of EUG shows remarkable anti-inflammatory, antioxidant, analgesic, and antimicrobial properties and has a significant effect on human health. The current manuscript summarizes the pharmacological characteristics of EUG and its potential health benefits.

## 1. Introduction

**Figure 1:**
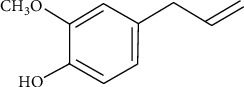
Structure of EUG.

Eugenol (EUG) or 4-allyl-2-methoxyphenol is a phenylpropanoid having an allyl chain-substituted guaiacol (Figure 1). EUG, a naturally occurring compound, has been reported to be present in several plant families including Holy basil or tulsi leaves (Lamiaceae), *Eugenia caryophyllata* (clove), *Zingiber officinale* (ginger), bark and leaves of *Cinnamomum verum* (cinnamon), *Curcuma longa* (turmeric), and peppers (Solanaceae) [1], as well as various aromatic plants such as *Cinnamomum verum* (true cinnamon), *Ocimum basilicum* (basil), *Myristica fragrans* Houtt. (nutmeg), and *Cinnamomum loureirii* Nees. (Saigon cinnamon). The major natural sources of EUG are *Eugenia caryophyllata* (syn *Syzygium aromaticum*) which comprises 45-90% [2], and cinnamon has 20-50% of EUG, but the commercial level extraction of EUG is quite expensive with longer cultivation times, while ginger, tulsi, and bay can be used instead of cinnamon and clove as cheaper source [1].

EUG can be prepared synthetically by guaiacol allylation with allyl chloride or produced through biotransformation process that involves microorganisms like *Escherichia coli*, *Corynebacterium* sp., and *Bacillus cereus* [3]. The pharmacological properties of EUG are numerous including antimicrobial, anti-inflammatory, analgesic, neuroprotective, antidiabetic, and antitumor activities that make it a versatile natural ingredient that helps in the prevention and cure of several disorders. The WHO declared EUG generally recognized as safe (GRAS) and a nonmutagenic substance. This naturally occurring molecule also has a large utilization in perfumery industry and food industry [4]. The antiseptic property made its use in mouthwashes as a disinfectant and also complexed with tooth fillers due to its pain relieving, antiseptic, and analgesic properties [5, 6]. In agricultural practices, EUG have extensively been used as an insect attractant and even in pesticide production. At commercial scale, EUG proven itself a versatile compound mainly due to its structure, complex formation, and an excellent substrate for carrying out biotranformations [7, 8].

Medicinally important plant species are a good source of active and potent drugs bearing extensive pharmacological properties. There is a huge list of pharmacological activities and bioactivities of EUG along with industrial utilization. In spite of its strong antioxidant properties, EUG protects neuron cells and recues the chances of the oxidative stress-oriented diseases as well as inflammatory diseases. Moreover, EUG is also used in local anesthesia to check the multiple pathological pains other than its wide utilization in dental clinics. Keeping in view the broad-spectrum utilization of EUG, the current review is aimed at highlighting and summarizing the recent advancements using EUG and exploring its pharmacological properties in a broader way. Moreover, this review will also discuss the roles of EUG in inflammatory and chronic diseases, in its antioxidant potential, and in neuroprotection.

## 2. Pharmacological Properties of EUG

The mechanism behind the therapeutic potential of EUG has been explained in huge literature. EUG is effective against a number of diseases such as reproductive disorders, nervous system disorders, blood glucose and cholesterol irregularities, microbial infections, tumorigenesis, hypertension, inflammations, and digestive complications [9]. Herein, the potential of EUG in combating severe illnesses and the mechanisms linked with health-promoting actions have been illustrated in detail [1].

### 2.1. Anticancer Activities

**Figure 2:**
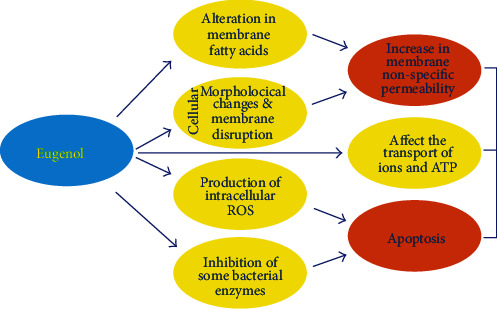
Cellular mechanism of EUG on cancer cells.

Cancer usually forms tumors by accumulation of cells and involves uncontrolled cell division. It is the second major cause of death worldwide, with a 6 million fatality rate annually [10, 11]. Cellular aggregation can be a consequence of inflammation because of inappropriate performance of signaling pathways [11]. Chemotherapy is mostly employed to destroy cancer cells, but in addition to targeting the diseased cells, it also causes division of normal cells of hair follicles, bone marrow, etc. Therefore, chemopreventive natural agents, like EUG, are preferred for tumor therapy. These drugs, even at high dose, show no cytotoxic effect on healthy cells [12–14]. EUG has been declared nonmutagenic and noncarcinogenic by the US Food and Drug Administration (FDA) [15–17] (Figure 2).

Different studies on EUG demonstrated its strong potential in combating colon cancer, prostate cancer, skin tumors, and gastric cancers [11, 18–20]. Therapeutic drugs undergo apoptosis as a therapy for cancer and many other diseases. Apoptosis is programmed cell death which causes plasma membrane shrinkage, blebbing, fragmentation in chromosomal DNA, production of membrane-bound small apoptotic bodies which are phagocytozed by nearby surrounding cells, and chromatin condensation [10, 21]. Apoptosis is a vital function of the human body without which there is surely a great risk of many disorders like cancers, acquired immune deficiency syndrome (AIDS), etc. [10, 11, 22].

Drug combination therapies are mostly used in combating cancer [23]. EUG shows a synergistic effect when used with some chemoinhibitory drug leading to a great reduction in drug toxicity on healthy cells [24]. In an *in vitro* study, use of little quantity of EUG in combination with gemcitabine potentiates the effects of the drug with no side effects on healthy cells [1, 11, 25].

Breast cancer is ranked second among most common cancers in women, and it is classified as the fourth common cause of cancer-linked deaths worldwide [1]. Mammary epithelial cells in women are regulated by maintaining a balance among the process of their proliferation and apoptosis [26]. Disturbance in this balance leads to a rise in mammary epithelial cells finally causing breast cancer [26]. Vidhya and Devaraj [27] proved that breast cancer cells (MCF-7) experience strong antimutagenic activity of EUG. EUG is both time and dose dependent when suppressing proliferation of MCF-7 cells [17, 27]. Pisano and colleagues [28] also explained the antiproliferative action of EUG-associated biphenyl (S)-6,60-dibromo-dehydrodieugenol, by initiating apoptosis.

Melanoma or malignant melanoma develops from melanocytes and is a sort of skin cancer [29]. Among all skin cancers, melanoma accounts for 4% only. However, it has a high mortality rate with over 80% of death toll from skin cancer [30]. The antiproliferative effect of EUG against melanocytes was studied by Pisano and coworkers [28]. EUG seize cell cycle and promote apoptosis. This effect of EUG has also been studied in a B16 xenograft model by Miyazawa and Hisama [31]. EUG acts by synthesis of ROS [17] which causes inhibition of DNA synthesis thus delaying tumor growth. 40% reduction was reported in tumor size by the action of EUG [2, 32]. Another study was conducted by considering the anticancer activity of EUG on human melanoma cells (WM1205Lu). The results indicated apoptosis and cell cycle arrest at S-phase [2]. Ghosh and coworkers [33] examined EUG and isoeugenol for antimelanoma activity. They inferred that EUG, but not isoeugenol (isomer of EUG), showed anticancer activity against melanocytes [17].

Cervical cancer arises from the cervix. In 2002, cervical cancer resulted in 274,000 deaths [34]. More than 90% of cervical cancer cases are caused by human papillomavirus infection; most people who have had HPV infections, however, do not develop cervical cancer [35, 36]. Experiments were conducted for studying the action of methyl EUG and cisplatin against cervical cancer cells (HeLa cells). The drugs were used separately and in combination. Methyl-EUG combined to cisplatin enhanced the anticancer effect by inducing apoptosis and destroying HeLa cells compared to the ample drug effects. Number of cells in G0/G1 phase, caspase-3 activity, and mitochondrial membrane potential loss were much enhanced in combination treatment in contrast to single drug exposure [23]. EUG showed a dose-dependent effect on HeLa cells [11].

### 2.2. Antioxidant Potential

**Figure 3:**
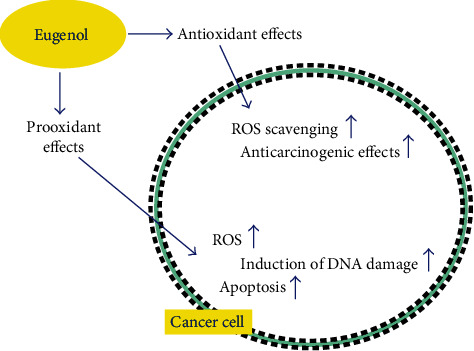
Prooxidant and antioxidant effects of EUG against cancer cells.

Antioxidants protect body from the harms of free radicals by eliminating ROS or scavenging free radicals [37–39]. Unnecessary groups of free radicals are responsible for multiple human diseases like AIDS, cancer, and Parkinson's disease [1]. For a healthy body system, free radical production must be depressed [40]. Phenolic groups play a vital role in antioxidant action [41]. Availability of electrons for neutralizing free radicals is the basic principle of antioxidant action. Increasing the number of hydroxyl groups in phenol ring increases the capability to behave as hydrogen donor and prohibits oxidation [40]. Oxidative stress is an imbalance in antioxidant defense and ROS production [42, 43]. Due to the disturbance in equilibrium between prooxidants and antioxidants, ROS production enhances leading oxidative stress which is concerned with inhibition of normal body functions [40]. Moreover, EUG has been reported to pose both prooxidant and antioxidant effects when applied to the cancer cells in a concentration-dependent fashion [44] (Figure 3). It was shown by electron spin resonance that the scavenging effect of EUG is due to the allyl group in its structure [39, 45]. It hinders in lipid peroxidation leading to free radical destruction [46]. The EUG have great inhibitory effects on hexanal oxidation [47], copper-dependent LDL oxidation, iron-mediated lipid peroxidation [39], and nonenzymatic peroxidation in liver mitochondria [46] and hinder the onset of oxidative stress-mediated diseases. EUG has an extraordinary reducing ability [48] and donates phenolic hydroxyl groups that can react with free radicals, diminishing oxidative stress thus a desirous antioxidant. Moreover, the antioxidant behaviour of EUG is greater than most of the known or standard antioxidants such as Trolox [40].

EUG shows dual properties, i.e., antioxidant as well as prooxidant actions, and later is the cause of cytotoxicity [49]. EUG behaves as an antioxidant at lower concentrations by minimizing ROS-mediated oxidative stress, but on the contrary, EUG at higher concentrations acts as a prooxidant to enhance the production of the ROS [16, 43, 50, 51]. Few studies showed that EUG eliminated free radicals through ABTS (2,2-azinobis (3-ethylbenzothiazoline-6-sulfonic acid) (76.9%) and DPPH (89.9%) in *L*-ascorbic acid [52]. Kaur and coworkers pretreated male Swiss albino mice with EUG and found a reduction in lipid peroxidation (LPO) and an inhibited depletion of antioxidant enzymes.

### 2.3. Cardiovascular Protection

Hyperlipidemia is the most common social issue in common people and causes cardiovascular diseases (CVDs) and lipid-related diseases [53]. CVDs and hyperlipidemic disorders are caused by less physical activity and high fatty acid intake [1]. High low-density lipoprotein cholesterol (LDL-c) leads to toxicity in vascular tissues and atherosclerosis/atherogenesis eventually causing diabetes; obesity; hypertension; inappropriate working of main body organs such as the kidneys, heart, and liver; and atherosclerosis [54].

CVD death and disease rate can be eradicated by less intake of lipids in the diet [55]. Reduced LDL-c concentrations also support in diminishing CVDs and improve atherosclerotic state [56, 57]. ROS causes an increase or imbalance in oxidative stress homeostasis which results in enhancing a number of chronic diseases like coronary heart disease (CHD). Increased ROS production causes oxidative damage due to the disturbance in cellular antioxidant status [58].

Dyslipidemia is another example which is linked with oxidative stress and causes disregulation in working of cellular antioxidant stress response system [59]. Rice bran is widely used since decades because of its cholesterol-lowering, free radical scavenging, and antiatherogenic actions [60].

However, it is preferable to use natural and safer drugs in as minimum quantities as possible. EUG has a great antihypercholesterolemic and antiatherogenic potential. Venkadeswaran et al. [61] reported hypolipidemic effects of EUG in intraperitoneally injected Triton WR-1339 (300 mg/kg per B.W.) induced hyperlipidemic Wister male rats. EUG was more effective against high lipid and cholesterol content in comparison to a lipid-lowering drug (lovastatin). It caused a 55.88% reduction in total cholesterol; also, LDL-C (79.48%) and triglycerides (64.30%) were reduced.

EUG undergoes dose- and endothelium-dependent reversible vasodilator responses [20]. In hypersensitive rats, EUG showed a hyposensitive behaviour by inducing vascular relaxation [62]. The smooth muscle relaxant action of EUG is due to its blocking action on receptor-operated and voltage-sensitive channels. Vascular relaxation is done via endothelial-generated nitric oxide (NO) [63]. The hypocholesterolemic action of EUG was well explained by a recent study using a hyperlipidemic zebra fish model, where EUG caused a great reduction in triglyceride (80%) and cholesterol (68%) levels in serum samples [64].

Hypercholesterolemia also causes an imbalance in the serum concentration of malondialdehyde (MDA, increased) and superoxide dismutase (SOD, decreased). This effect was studied by Munisa et al. [65], by the application of EUG against MDA and SOD in rats with high cholesterol level. They concluded that EUG regressed the concentrations to their normal ranges. Eugenol exerted negative inotropic effects in guinea pig heart muscles [66]. According to Choudhary et al. [67] EUG hinders isoproterenol-induced cardiac hypertrophy. The study was conducted on male Wister rats against EUG. They concluded that apoptosis, isoproterenol-induced oxidative stress, and calcineurin action in serum were suppressed [20].

### 2.4. Antidiabetic Activity

Hyperglycemia is a condition after which there is an outbreak of a degenerative disease called diabetes mellitus caused by abnormalities in glucose metabolism. Studies have shown that EUG is capable of curing several metabolic illnesses owing to its various pharmacological activities. EUG is a strong antidiabetic bioactive substance [1]. According to Anuj and Sanjay [39], EUG principally diminishes *α*-glucosidases after which it intercepts the development of advanced glycation end (AGE) products [68]. In the presence of inhibitory *α*-glycosidase compounds like EUG, dietetic complex carbohydrates do not undergo liberation of absorbable monosaccharides. The inhibitors prevent any drastic rise in meal-induced glucose level in blood by delaying the absorption of glucose into the bloodstream [69].

EUG enhances the activities of carbohydrate metabolism enzymes like glucose-6-phosphate dehydrogenase (31.05%) and instance hexokinase (62.25%). Studies show that a diminishing effect has also been observed by the application of EUG on creatine kinase (38.57%), glucose-6-phosphatase (24.45%), blood urea nitrogen (34.01%), and fructose-1,6-bisphosphatase [70].

Tahir et al. [68] demonstrated the antidiabetic potential of S. *aromaticum* and *Cuminum cyminum* (*C. cyminum*) following the *α*-amylase enzyme assay method. Starch is converted to simple sugars by *α*-amylase which is a human enzyme and is involved in causing diabetes. The rate at which glucose absorbs into the blood stream can be reduced by the application of inhibition of *α*-amylase enzyme. The enzyme retards the digestion of carbohydrates ultimately causing a decrease in blood glucose level. The antidiabetic activity is dose dependent. *S. aromaticum* depicted more antidiabetic potential than *C. cyminum*, as *S. aromaticum* is chiefly composed of EUG (18.7%) while the main constituent of *C. cyminum* is composed of *á*-pinene (18.8%) [68]; the antidiabetic potential of *S. aromaticum* was found to be 95.30% which is greater than 83.09% of *C. cyminum*. These results proved that EUG has a great antidiabetic potential compared with many related essential oils [68].

Srinivasan et al. experimented male diabetic rats by inducing streptozotocin drug (40 mg/kg per B.W.) in them. The activity of major enzymes taking part in the glucose metabolism was studied to examine efficacy of EUG. In contrast, only a 10 mg/kg per B.W. dosage of EUG greatly enhanced hepatic glycogen and insulin in plasma (46.15%) samples with a reduction in glycosylated haemoglobin (25.70%) and blood glucose (70%) [70].

### 2.5. Antiparasite Activity

The majority of human parasites are a key cause of increased mortalities compared with rest of the issues excluding tuberculosis (TB) and AIDS [71]. Certain drastic effects of EUG have been reported on morphology and growth of various parasites like *Trypanosoma cruzi*, *Giardia lamblia*, and *Leishmania donovani* [72]. Schistosomiasis is a parasitic helminthic infection in about 78 different countries caused by parasitic flatworms or blood fluke schistosomes (*S. haematobium*, *S. mansoni*, *S*. *japonicum*, and S. *mansoni*) and also familiar as bilharzia or snail fever which is somewhat a neglected tropical disease [72–75]. Schistosomiasis gets settled in intestinal venules to affect the gut, urinary tract, and liver which results in retardation of growth, anemia, enlarged liver, and even liver fibrosis [72]. EUG can be used as an antiparasitic agent with least side effects than other chemotherapeutics in curing various parasitic diseases, and overdose of EUG shows antileishmanial, antimalarial, and antihelmintic potentials. In an *in vivo* study using mouse models, the drug-resistant parasite *Schistosoma mansoni* showed a great reduction (19.2%) following a synergistic effect of EUG in combination with conventional drugs [72]. Moreover, it is further noticed that EUG had no effect on egg development of worms owing to the fact that the oogram patterns of the treated group of mice and nontreated group of mice were similar, but the egg density in the walls of the intestines of mice was greatly reduced by the application of EUG [76]. Protozoans like *Leishmania* are the major cause of leishmaniasis in humans, and the pathogens are being transferred through the bite of infected phlebotomine female sandflies [77]. Leishmaniasis can be mucosal, visceral, or cutaneous and if it gets combined with human immunodeficiency virus may cause a serious condition [39]. Most of the pathogenic parasites have developed resistance with course of time against standard drugs; thus, use of natural phytochemicals like EUG is preferred [78]. Following a 60 min treatment of EUG in combination with essential oils showed absolute (100%) destruction of L. amazonensis parasites [79] primarily by the increased production of EUG-mediated nitric oxide (NO) which is an antileishmanial oxidant involved in triggering the killing of leishmania species [39, 79].

### 2.6. Antibacterial and Antiviral Activities

EUG have been well known for its antimicrobial potential and uses. Majority of food-borne diseases are attributed to the microorganisms and are an escalating health issue of global public. About one-third of the population in industrial countries are facing food-borne diseases issues. In the US, 31 notable pathogens give rise to approximately 9.4 million cases of food-borne diseases per annum [80]. EUG can be bactericidal (bacterial killing) or bacteriostatic (inhibiting bacterial growth) depending on the MIC (minimum inhibitory concentration) and MBC (minimum bactericidal concentration) [81] and have antivirus and antifungal effects at the same time. EUG shows dynamic antibacterial ability against a variety of strains of gram-negative (*Pseudomonas aeruginosa*, *Salmonella choleraesuis*, *Yersinia enterocolitica*, *Helicobacter pylori*, and *E. coli*) and gram-positive (*Streptococcus pneumonia*, *S. aureus*, *Enterococcus faecalis*, and *Streptococcus pyogenes*) bacterial strains. EUG has proven to be active against several bacterial enzymes like histidine, amylase, ATPase, and proteases [82, 83]. The presence of a free hydroxyl group in the EUG molecule is the main cause of its great antimicrobial activity [84]. EUG extracted from clove oil adversely disrupts the cell wall and cell membrane of bacteria, and this lysis leads to an enhanced fluidity and permeability of cell membrane [85, 86] followed by membrane expansion, leakage of intracellular fluids accompanied by proteins and lipids release, respiration inhibition, alteration of ion transport patterns of bacteria [81, 83], and disturbance of membrane-embedded proteins, consequently leading to the cell death [87]. Catherine et al. [88] tested the antibacterial potential of EUG in combating (MIC 0.15-0.25%) food-borne bacteria *E. coli*, *Yersinia enterocolitica*, *S. aureus*, and *Bacillus cereus* and proven that gram-negative bacteria are more resistant than its counterpart gram positive [88]. The combination therapy by making fusion of plants is quite effective for the treatment of critical bacterial infections [2, 89]. The antibacterial activity of EUG was amplified when combined with vancomycin, where the half of the bacterial membranes are damaged due to EUG and hence facilitate the vancomycin penetration into the membrane [2, 90]. The combined effect of EUG with gentamicin or ampicillin resulted in a much higher killing rate in an hour time than EUG applied alone [2, 91].

Herpes simplex virus (HSV) infects persons 50 years and is a frequent sexually transmitted infection [92]. The medicinal plants are a rich source of EUG that retards the replication of HSV by neutralizing and inactivating the viral infections [93, 94]. The mechanism behind antiviral actions of EUG is the upregulation of the expression of HSV-1-glycoprotein B that checks HSV replication [95], while some studies confirmed that EUG inhibits the replication of the HSV-1 and HSV-2 DNA molecules, as well as damages the outer envelope of newly synthesized virions [96–98]. The benefits of using combination chemotherapy includes enhanced antiviral activity, reduced dosage of toxic substances, and lessened development of drug resistance [99]. In in vivo study involving mouse models, *Eugenia caryophyllus* extracts rich in EUG which were applied in combination with acyclovir (ACV) showed an anti-HSV-1 potential in the brain and skins of mice [97, 100]. In this ACV-EUG therapy, ACV specifically targets DNA molecules and inhibits the replication process [101]. In HSV-1-infected mice, EUG therapy delayed the production of herpetic keratitis in the cornea [39] which shows a close association of EUG on the ocular herpetic infections [93]. EUG disintegrates the lipidic envelope of the HSV virus and also induces glutathione S-transferase (GST) expression in liver cells of rats, eventually blocking replication process.

### 2.7. Antifungal Activity

EUG has shown lethal effects on the growth of different strains of fungi such as *Fusarium graminearum*, *F. moniliforme*, *Tricophyton rubrum*, *Penicillium citrinum*, *Candida tropicalis*, *C. krusei*, and *Aspergillus ochraceus* . EUG either causes cell cycle arrest or affects fungal cell membrane integrity by disruption [102]. Owing to the lipophilic nature of the EUG, it gets accumulated in the phospholipid bilayer of fungal cells, altering the functions of vital membrane-bounded enzymes, affecting permeability, fluidity of membrane, and envelope morphogenesis [103, 104]. Another recent study claimed that by the addition of the methyl radical in the EUG, it may amplify its antifungal potent to many folds [105]. Combination therapies are often employed for better results. A synergistic interaction of EUG or isoeugenol with that of fluconazole (EUG/isoeugenol-fluconazole/amphotericin B) was applied against multidrug-resistant strain *C. albicans* and found extraordinary effectiveness [2]. Furthermore, EUG have been reported to affect the morphology of the fungal strains especially the hyphae of *B. cinerea* which showed much irregular structural features such as broken or shriveled hyphae, large vesicles, and leaked intracellular contents. In addition to morphological alterations, EUG induces the accumulation of ROS especially hydrogen peroxide (H_2_O_2_) in the fungal cells to generate oxidative stress which ultimately bursts the fungal hyphae [103].

### 2.8. Anti-Inflammatory Activity

**Figure 4:**
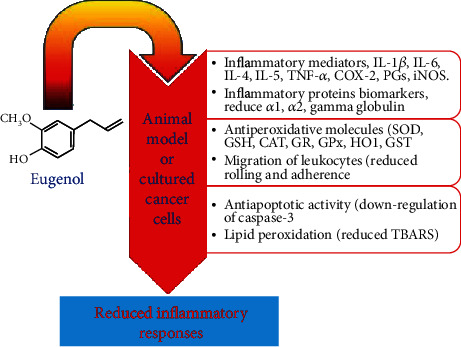
Effect of EUG on controlling the inflammations.

Inflammation is called as the body's adaptive immunity response, triggered by noxious stimuli, tissue infection, and injury [106]. It can be chronic or acute [107], and anti-inflammatory drugs used nowadays have adverse effects [108]. EUG, with no side effects, has a tremendous anti-inflammatory potential (Figure 4). EUG can be used in the protection of damage resulting from oxidative stress [52]. The ROS-mediated oxidative stress results in cellular damage and LPO [109]. Saraiva et al. [108] showed that oxidative stress and inflammation are interlinked mechanisms. In a study, male Swiss albino mice when treated with EUG have undergone reduced LOP and in expression levels of inflammatory markers, viz., COX-2, iNOS, and cytokine tumor necrosis factor *α* (TNF-*α*), and antioxidant enzymes [19]. Generally, EUG pretreatment reduces inflammations (as a result of the action of LPS on lungs) in cells.

Biswas et al. [110] studied pulmonary inflammation in mouse and action of EUG as an antiagent. It was concluded that EUG causes significant reduction in the much higher levels of neutrophils and TNF-*α* and also maintained the proinflammatory mediators. Magalhães et al. [111] investigated anti-inflammatory properties of EUG in lipopolysaccharide-induced lung injury for 6 h *in vivo*. A decrease of proinflammatory cytokines like TNF-*α*, NF-*κ*B-mediated signalizing pathway, and macrophage infiltration were reported, eventually leading to an improvement in the function and structure of the lungs due to a decrease in inflammation [52]. EUG inhibits TNF-*α* and cyclooxygenase-2 (COX-2) expression [112]. Its anti-inflammatory action is attributed to macrophage chemotaxis and prostaglandin synthesis. In a study, it was revealed that EUG suppresses the activation of NF-*κ*B-stimulating macrophages mainly due to TNF-*α* and COX-2 actions. COX-2 effect is enhanced by lipopolysaccharides (LPS) [113]. The expression of antioxidant enzymes like glutathione peroxidase (GPX) and superoxide dismutase (SOD) is significantly enhanced by EUG (Figure 4).

### 2.9. Antipyretic Activity

The antipyretic efficacy of EUG was examined by Feng and coworkers [114], where they found an antipyretic effect of EUG against a well-known antipyretic acetaminophen in rabbits made febrile by IL-1*β*. It shows a significant antifever action than acetaminophen when induced intragastrically, centrally, and intravenously. When given intragastrically, EUG in addition to treating fever, induces slight hypothermia unlike acetaminophen. Acetaminophen reduced fever by 68% at a dose of 1.3 mM/kg while EUG reduced 68% fever at a much less dosage. When given peripherally, it shows marked results in inhibiting fever. When given intravenously, respiration of rabbits and vasodilation in ears were increased. Increased respiration depicts that EUG have a positive action on the central nervous system (CNS). If prostaglandins and sodium archidonate mediate fever in the CNS, acetaminophen and EUG can inhibit fever by retardation of prostaglandin R and sodium archidonate synthesis. Both these drugs show the same antipyretic mechanism but EUG shows more potent behaviour as compared to acetaminophen [115].

### 2.10. Antinociceptive Activity (Analgesic)

EUG is significantly efficient in relieving pain by reducing the responses that are linked with pain. It suppresses several responses to histamine, norepinephrine, and stimulation of periarterial sympathetic nerves [116] and inhibits prostaglandin synthesis [117]. It is mostly used as a dental analgesic and antiseptic agent [118]. Its analgesic effect is linked to suppress Na^+^, K^+^, and Ca^2+^ voltage-dependent channels [119, 120]. The high-voltage-activated Ca^2+^ channel (HVACC) inhibition by EUG plays an important role in pain-relieving effect. It inhibited HVACC currents in both capsaicin-insensitive and capsaicin-sensitive dental primary afferent neurons [121]. TRPV-1 receptors are involved in pain stimulation. A study revealed that EUG inhibited TRPV-1 by suppressing voltage-activated Na^+^ and Ca^2+^ channels [2, 122].

EUG is an antagonist towards NMDA (N-methyl-D-aspartate) and gamma-aminobutyric acid (GABA); both are involved in pain transmission [123, 124]. It inhibits Ca^2+^-dependent release of neurotransmitters and also retards interleukin (IL-)1*β* and PGE2 synthesis [39]. Park et al. [125] studied the analgesic action of EUG in the orofacial part and reported its inhibitory action on VGSCs in trigeminal ganglion (TG) neurons.

Ferland et al. [126] performed experiments on a monoiodoacetate-induced rat model of osteoarthritis to find the effects of EUG. The affected limb showed marked improvement when administered with EUG for two days after osteoarthritis induction. Also, calcitonin gene-related peptide (CGRP) which is associated with spinal cord pain was reduced. The antinociceptive potential of EUG was investigated by Daniel et al. [127] in mice using acetic-acid-induced abdominal writhing process. EUG showed a significant result in decreasing the pain. The effect of EUG in several experimental pain models using mice was studied such as acetic acid-mediated abdominal constrictions, formalin-mediated hyperalgesia, and thermal pains, and 92.73%, 70.33%, and mild inhibitions in pain were found, respectively [128].

### 2.11. Effects on the Central Nervous System (CNS), Neuroprotection, and Antistress Activity

**Figure 5:**
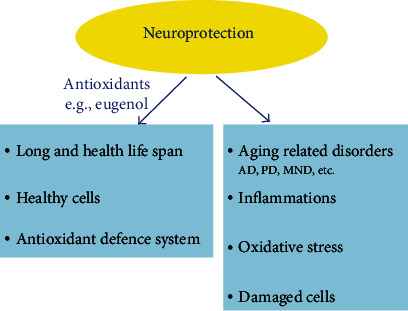
The role of EUG in neuroprotection and neurodegenerative diseases in the CNS involves various mechanisms including oxidative stress, damaged/unrepaired cells, and inflammations.

EUG in addition to acting on the periphery also performs actions in the central nervous system (Figure 5). The hydrophobic property of EUG makes it efficient in penetrating the blood-brain barrier for its entry into the brain and performs its action *in vivo* [129]. EUG guards neuronal cells against N-methyl-D-aspartate- (NDMA-) induced oxidative and excitotoxic injury [39]. EUG shows a neuroprotective potential on hippocampal tissues owing to its power reduce brain-derived neurotrophic factor (BDNF), and retardation of amyloid-*β* peptide (A-*β*) induced cell death through the abnormal blockage of Ca^2+^ (resulted from A-*β*) [130]. Its ability to restrict antioxidant and excitotoxicity effects also contributes in neuroprotection. In cell models, EUG improves actions of few glutathione-related proteins and shields essential neuronal cells from oxidative and excitotoxic effects [131]. The inhibitory action of EUG has been reported on 5-lipoxygenase, in addition to an improved action in response to excitotoxic and ROS-injured neuron cells [132].

Depression, a neurodegenerative disorder, causes common psychological disorders, and 10-20% of the general population suffer through it. EUG showed a marked effect as an antidepressant in force swimming test (FST) and tail suspension test (TST) comparable to an antistress drug imipramine [39]. The mechanism of action of these two drugs is different. Real-time PCR (RT-PCR) showed that metallothionein-III (MT-III) was found to be linked with the antidepressive effect of EUG quite different from imipramine. This forms the basis of an alternative treatment if the patients develop resistance to typical drugs. EUG exhibits antiepileptic potential. It performs its action by blocking long-term potentiating and synaptic transmission in neurons [133]. Sen et al. [134] reported the antistress activity of EUG. EUG acts at a central level to pose neuroprotective effects against ischemia, excitotoxicity, and amyloid-*β* peptide. In experimental diabetes, EUG improves vascular and neuronal complications and suppresses the transmission of action potential in sciatic nerves [39]. It also elongates the kallikrein- and bradykinin-induced tranquilization [135]. EUG is a good medicine for Alzheimer's disease (AD) and depression. Moreover, the expression of the BDNF gene in the hippocampus is also controlled by EUG. It also retards monoamine oxidase A (MAO-A) and sometimes reverts back monoamines that are reduced in the brains of depressed patients [129]. EUG has been proved useful against stress-induced irritable bowel syndrome (IBS) [136].

Regulation of release of neurotransmitters by EUG eventually moderates different brain functions. Rats with 4 h restraint stress model were studied by Garabadu et al. [137], and antistress potential of EUG was observed. It was concluded that EUG administration enhanced ulcer index and corticosterone and norepinephrine levels. EUG also brought alteration in serotonin (5-HT) levels in almost all parts of the brain. This study elaborates the antistress actions of EUG that are ascribed with the modulation of hypothalamic pituitary adrenal (HPA) as well as brain monoaminergic systems (BMS). Under stress situations, sympathoadrenal (SA) and BMS and HPA regulate several physiological and psychological responses [137].

The neuroprotective efficacy of EUG and isoeugenol in response to acrylamide-mediated neuropathy in male albino rats was studied by Prasad [138], where it was found that both the drugs increase the behavioral index gait level and reduce the oxidative stress-linked markers like NO and ROS. Dopamine, acetylcholinesterase activity, and cytosolic Ca^2+^ levels are also diminished in the brain. It is further demonstrated that EUG and isoeugenol potentially restrict acrylamide-mediated neuropathic condition in rats and due to this factor, they can be directly used through diet to soothe different neuropathic conditions in humans [138].

### 2.12. Neurodegenerative Disorders

Neurological disorders include a number of conditions whose inception results from subsequent degradation of normal function and morphology of neurons, mainly in the CNS [139]. Globally, 20 million people are suffering from only these three disorders [140]. Neural disorders have been ranked by the WHO as the second (12%) most reason of mortalities worldwide. Disorders linked with neurodegeneration include Parkinson's disease (PD), AD, prion diseases, ischemia, hydrocephalus, Huntington's disease, head and brain malformations, and spongiform encephalopathies [141]. Among the neurodegenerative diseases, AD, PD, and motor neuron disease (MND) are most common. Natural agents are frequently employed for the treatment of various neurological disorders (Figure 5). It was demonstrated by ancient Chinese researchers that a number of herbs used in treatment of AD consists of *Rhizoma acori graminei* extracts enriched with EUG [39].

The neurodegenerative disorder AD is described characterized by the loss of neurotransmitters and neurons and is accompanied by depression and PD [129]. Neuronal degeneration in the CNS involves various mechanisms including apoptosis, protein accumulation, ageing, and oxidative stress, which are contributing to neurodegenerative disorders (Figure 5). The most common among these is the aggregation of misfolded proteins, which display amyloid characteristic (formation of *β*-pleated sheets), calling this group of illness called as brain amyloidosis [140].

### 2.13. The Enhancement in Skin Permeation

The stratum corneum of the skin is composed in a way to control the passive movement of substances across the skin. A common way for enhancing drug permeation through the skin is the transdermal drug delivery. Use of small electric charge for the transport of a substance (drug) across the skin is called iontophoresis and is used to compensate for impermeability of the skin, in addition to chemical permeation enhancers like essential oils [142, 143]. Anuj and Sanjay [39] investigated the permeation enhancer ability of EUG in the absorption of tamoxifen. EUG increased the permeability of tamoxifen across porcine epidermis as compared to 50% ethanol. Furthermore, EUG and acetyl-eugenol were also seen for transdermal delivery of ibuprofen in rabbits and noted to enhance permeation with great 7.3 enhancement ratio [144].

### 2.14. Toxicity

EUG toxic behaviour has been studied in multiple *in vivo* studies. However, little or no information is available in humans [39]. EUG toxic behaviour depends mainly on its concentration; i.e., its effects are dose dependent [52]. The prooxidant effect of EUG leads to its toxicity [43], and Medeiros et al., [145] reported that toxicity of EUG is attributed to protein inactivation due to the binding of EUG at the lysine residues. Cytotoxicity of EUG is possibly due to its metabolic reactions. The reactive metabolites then react further with DNA, forming adducts that can destroy nuclear genetic material. EUG has been proved as a contact allergen in dentistry [39] and makes its entry into the bloodstream by penetrating dental pulp tissue, causing chromosomal aberrations (CAs) in dental pulp cells in humans [146].

### 2.15. Wide Application of EUG in Dentistry

EUG, due to its antioxidant, anti-inflammatory, anesthetic, and analgesic potentials, is used extensively in dentistry at low dosage. In dental emergencies, it is used as an anodyne [147]. It is used as a cementing material in dentistry [118], and the first type of EUG cement was developed earlier back in 1933 which comprises of EUG liquid and ZnO powder, forming a paste. In tooth preparation, EUG owing to its sedative potential provides soothing action for assisting in pulp relaxation after contusion [148]. ZnO EUG cement is presently being used in dentistry in root canal sealing; temporary fillings and indirect pulp capping are the widespread uses of ZnO+EUG cement (ZOE) [147]. Park et al. [125] studied the action of EUG on pain behaviors in the orofacial region and the retardation of voltage-gated sodium channels (VGSCs) in trigeminal ganglion (TG) neuron cells.

### 2.16. Miscellaneous

At high concentrations, EUG has also been reported to provide anesthetic effects during a pharmacokinetic study using male Sprague-Dawley rats [149]. Tajuddin et al. [150] observed an improved sexual behaviour of male Swiss mice after EUG administration. It also shows antiulcerogenic effects. Furthermore, two ulcerogenic agents induced gastric ulcer which was diminished by treatment with EUG. The gravity of lesions was also decreased by EUG [151]. The antiulcer action of EUG is brought about by its free radical scavenging activity, less acid-pepsin secretion, inhibition of a great rise in NO level, and opening of ATP-sensitive potassium (KATP) channels [53]. *In vivo* examination finds that diarrhea caused by castor oil was reduced by EUG. Also, the intestinal aggregation of fluid induced by PGE2, the rate of intestinal transit, and the tone of isolated gut muscle and myometrium were also suppressed [152]. Karmakar et al. [153] reported an ovariectomised (OVX) model of rats induced with osteoporosis against EUG and its derivatives. The drug showed a marked effect on the efficiency of bone preservation.

## 3. Conclusion

The current review summarized the potential health benefits and effectiveness of EUG as a therapeutic agent which can be used in medicines and food for the treatment of inflammatory and oxidative stress-oriented disorders. The antioxidant, anti-inflammatory, antipyretic, analgesic, antiparasite, and antimicrobial properties of EUG are well described. It has a great role in neuroprotection, enhances skin permeability, relieves pains, and has a role in temporary dental filler formation (ZnO+EUG). EUG has no known toxicity in smaller quantities, but at higher concentrations, it behaves as prooxidant; hence, a strong anticancer activity is shown by this molecule. Furthermore, diverse applications of EUG such as its pharmacological importance in regulating blood cholesterol and lipid levels are also discussed. Future studies involving a specified dose range of EUG to cure different ailments are recommended to highlight this molecule for the development of drugs.

## Data Availability

All of the data used to elaborate and explain the findings herein are already given within the draft.

## Abbreviations

5-HT: 5-Hydroxytryptamine (serotonin)
ABTS: 2,2-Azinobis (3-ethylbenzothiazoline-6-sulfonic acid)
ACV: Acyclovir
AD: Alzheimer's disease
AGE: Advanced glycation end
AIDS: Acquired immune deficiency syndrome
A-*β*: Amyloid-*β* peptide
BDN: Brain-derived neurotrophic factor
BMS: Brain monoaminergic systems
CAs: Chromosomal aberrations
CGRP: Calcitonin gene-related peptide
CHD: Coronary heart disease
CNS: Central nervous system
COX-2: Cyclooxygenase-2
CVD: Cardiovascular diseases
DNA: Deoxyribonucleic acid
DPPH: 2,2-Diphenyl-1-picrylhydrazyl
EUG: Eugenol
FAO: Food and Agricultural Organization (of United Nations)
FDA: Food and Drug Administration
FST: Force swimming test
GABA: Gamma-aminobutyric acid
GPX: Glutathione peroxidase
GRAS: Generally recognized as safe
GST: Glutathione S-transferase
H_2_O_2_: Hydrogen peroxide
HPA: Hypothalamic pituitary adrenal
HSV: Herpes simplex virus
HVACC: High-voltage-activated Ca^2+^ channel
IL-1*β*: Interleukin-1*β*
IL-6: Interleukin-6
iNOS: Inducible nitric oxide synthase
KATP: ATP-sensitive potassium channels
LDL: Low-density lipoprotein
LDL-c: Low-density lipoprotein cholesterol
LPO: Lipid peroxidation
LPS: Lipopolysaccharides
MAO-A: Monoamine oxidase a
MBC: Minimum bactericidal concentration
MCF-7: Michigan Cancer Foundation-7
MDA: Malondialdehyde
MIC: Minimum inhibitory concentration
MND: Motor neuron disease
MT-III: Metallothionein-III
NF-*κ*B: Nuclear factor kappa light chain enhancer of activated beta cells
NO: Nitric oxide
OVX: Ovariectomised
PCR: Polymerase chain reaction
PD: Parkinson's disease
PGE2: Prostaglandin E_2_
ROS: Reactive oxygen species
SA: Sympathoadrenal
SOD: Superoxide dismutase
TB: Tuberculosis
TG: Trigeminal ganglion
TNF-*α*: Tumor necrosis factor *α*
TRPV-1: Transient receptor potential vanilloid-1
TST: Tail suspension test
VGSCs: Voltage-gated sodium channels
WHO: World Health Organization
ZnO: Zinc oxide
ZOE: Zinc-oxide eugenol.
